# Retraction: The AhR–TLR4 axis in non-IgE-mediated cow’s milk allergy: a systematic review with integrated multi-omics corroboration

**DOI:** 10.3389/falgy.2026.2026345

**Published:** 2026-10-16

**Authors:** Frontiers Editorial Office

The journal retracts the 14th April 2026 article cited above.

Following publication, concerns were raised regarding data integrity and the availability of the supporting datasets. An investigation conducted in accordance with Frontiers policies determined that the accession numbers provided in the article were invalid, and the reported data could not be substantiated.

Consequently, the reliability of the article's findings cannot be confirmed. Frontiers has therefore lost confidence in the article and retracted it.

This retraction was approved by the Chief Executive Editor of Frontiers. The authors received a communication regarding the retraction and had a chance to respond. This communication is recorded by the publisher.

